# Comparison of Microbiological and Probiotic Characteristics of Lactobacilli Isolates from Dairy Food Products and Animal Rumen Contents

**DOI:** 10.3390/microorganisms3020198

**Published:** 2015-04-15

**Authors:** Neethu Maria Jose, Craig R. Bunt, Malik Altaf Hussain

**Affiliations:** 1 Department of Wine, Food and Molecular Biosciences, Lincoln University, Lincoln 7647, New Zealand; E-Mail: neethumaria@rocketmail.com; 2 Department of Agriculture, Lincoln University, Lincoln 7647, New Zealand; E-Mail: craig.bunt@lincoln.ac.nz

**Keywords:** lactobacilli, dairy food, animal rumen, screening, comparing *in vitro* characteristics

## Abstract

Lactobacilli are employed in probiotic food preparations and as feed additives in poultry and livestock, due to health benefits associated with their consumption. The objective of this study was to evaluate and compare the probiotic potential of ten lactobacilli strains isolated from commercial dairy food products and animal rumen contents in New Zealand. Genetic identification of the isolates revealed that all belonged to the genus *Lactobacillus*, specifically the species *L.* *reuteri*, *L. rhamnosus* and *L.* *plantarum*. All isolates did not show any haemolytic behaviour. Isolates of dairy origin showed better tolerance to low pH stress. On the other hand, rumen isolates exhibited a higher tolerance to presence of bile salts. All isolates exhibited resistance to aminoglycoside antibiotics, however most were sensitive to ampicillin. Isolates of rumen origin demonstrated a higher inhibitory effect on *Listeria monocytogenes*, *Enterobacter aerogenes* and *Salmonella menston*. Bacterial adherence of all isolates increased with a decrease in pH. This screening study on lactobacilli isolates has assessed and identified potential probiotic candidates for further evaluation.

## 1. Introduction

It has recently been reported that global probiotics demand was worth USD 27.9 billion in 2011 and is expected to reach USD 44.9 billion in 2018 [1]. The global market for probiotics is mainly driven by high demand for probiotic yoghurt and growing consumption of functional foods. This report identified growing consumer awareness regarding gut health having played a key role in sustaining this market.

Lactobacilli form the major group of bacteria incorporated into foods for use as probiotics or functional foods. Amongst the lactic-acid producing group, species of *Lactobacillus*, *Bifidobacterium* and *Enterococcus*, such as *L. acidophilus*, *L. casei*, *L. reuteri*, *L. rhamnosus*, *L. lactis*, *L. crispatus*, *L. gasseri*, *B. animalis*, *B. bifidum*, *B. infantis*, *B. lactis*, *E. faecalis*, and *E. faecium*, are prevalent [2,3]. The non-lactic acid producers include *Bacillus cereus* and *B. subtilis*. In addition, yeast such as *Saccharomyces boulardii* and *S. cerevisiae* are also used as probiotics [4,5,6]. Probiotics are commercially available in the form of powder, liquid, gel, paste, granules or even as capsules, sachets, *etc.* [7,8]. To observe a positive health benefit from consumption, a minimum level of microorganisms is required: this level depends on the strain used and the required health benefit. The dose recommended is usually between 10^9^ and 10^11^ CFU/day [9].

An effective probiotic is expected to function and survive under a variety of physiological conditions. Screening factors for probiotic abilities, carried out in this study, were based on the following assumptions. Upon consumption, probiotic bacteria should survive transit in the gastro-intestinal tract where it is open to challenges, such as the low pH environment of stomach and bile salts of the upper intestinal tract [10,11,12,13]. Stomach pH can vary from as low as 1–2 under fasting conditions and up to 4–5 following ingestion of a meal [14,15]. Probiotics must also be safe, for example probiotic bacteria should not cause lysis of red blood cells. Furthermore, antibiotic resistance may be necessary for survival in the presence of co-administered drugs [9]. The genes conferring resistance in probiotics should be innate in nature and non-transferable to other bacteria [16]. Display of antimicrobial activity against common intestinal pathogens is also highly preferred [11]. A common mechanism of lactobacilli to achieve these activities towards pathogens is by the production of organic acids, which lowers the pH, thereby creating a hostile environment for the growth of other bacteria. Simultaneously, these organic acids can prove toxic to other bacteria, thereby inhibiting pathogen growth. Competitive inhibition for mucosal binding sites between pathogen and probiotic bacteria also limits the growth and colonization of pathogens in the body [17]. Probiotic bacteria must also be capable of adhering to intestinal epithelial lining in order to provide benefits in the host. Adherence enables the probiotic bacteria to persist for a longer time in the gut and enhances the host-bacteria interactions [18]. Adherence of probiotic bacteria also helps it to overcome peristalsis activity of stomach [19]. For this purpose, their surface properties were studied by performing the BATH (Bacterial Adherence to Hydrocarbons) test.

This study was undertaken to characterize and draw a comparison of potential probiotic characteristics of dairy *versus* rumen isolates. Dairy isolates are capable of growing in the food processing conditions whereas rumen isolates are well adapted to grow in the gastro-intestinal tract (GIT) environment. The objective was to see the performance characteristics exhibited by lactobacilli isolated from two diverse environments and make a comparison of their potential probiotic properties. There have been previous papers describing screening characteristics of potential probiotic lactobacilli from diverse sources such as traditional dairy food, swine origin, cheese, infant gut micro-biota, *etc.* [20,21,22,23]. However, this is perhaps the first study reporting a comparison of *in vitro* characteristics of strains isolated from commercial foods and environmental sources from New Zealand.

## 2. Experimental Section

### 2.1. Isolation, Phenotypic Characterization and Carbohydrate Fermentation Profile of Strains

The lactic acid bacteria (LAB) isolates used in this study were isolated from two different sources-commercial dairy food products and animal rumen contents in New Zealand. Dairy food products included yoghurt and two different types of cheeses. Rumen contents used in this study were obtained from cow. For all the four samples, 10 g or 10 mL of sample was added to 40 mL of de Man, Rogosa, Sharpe (MRS) broth (Oxoid, Basingstoke, UK) and homogenized by vortex mixing. The inoculated broth samples were incubated at 37 °C for 24 h under anaerobic conditions. Tubes showing turbidity were selected and inoculated onto MRS agar plates. Cultures were purified by re-streaking on MRS agar 2–3 times. The isolates were stored in 50% glycerol at −20 °C until further use.

The cultures were characterized as LAB by gram-staining and microscopic observation (using Nikon Eclipse 50i). Also colony morphology was studied by growing cultures on MRS agar plates. Oxidase activity was identified using oxidase colour indicating strips (Oxoid Microbact™ Identification kit, UK). Carbohydrate fermentation profiles of the isolates were generated according to the method described by Gupta *et al.* [24]. Sugars used to generate fermentation profiles included arabinose, cellobiose, fructose, glucose, galactose, lactose, mannose, mannitol, melibiose, maltose, raffinose, ribose, sorbitol and sucrose. Glycerol was used as a negative control.

### 2.2. Identification of LAB

DNA of the ten isolates was extracted using Gentra Puregene cell kit. Polymerase Chain Reaction (PCR) analysis of 16S–23S rRNA gene (intergenic spacer region) of *Lactobacillus* isolates using primers 5′-GAATCGCTAGTAATCG-3′ and 3′-GGGTTCCCCCATTCGGA-5′ was performed followed by agarose gel electrophoresis. Amplified PCR products were sequenced by the Bio-Protection Research Centre (Christchurch, New Zealand). The sequences obtained were analysed using the nucleotide blast program provided by the online Basic Local Alignment Search Tool (BLAST^®^), a database search tool, developed and maintained by the National Center for Biotechnology Information (NCBI) available online at http://blast.ncbi.nlm.nih.gov/Blast.cgi.

### 2.3. Acid Tolerance

Overnight cultures of lactobacilli strains were added to MRS broth adjusted to pH 2 and pH 3, with 1 M HCl. The initial bacterial concentration was 10^6^ CFU/mL. The broths were incubated for 6 h and cell viability was determined by serial dilution and plating onto MRS agar after 0, 3 and 6 h incubation.

### 2.4. Bile Salt Tolerance

To determine bile salt tolerance strains were grown overnight in MRS broth. Sufficient cell suspension to give 10^6^ CFU/mL concentration of each isolate was added into 10 mL of fresh MRS media containing 0.3% and 2% of bile salts (Oxoid, UK). The broths were incubated for 6 h and cell viability was determined by serial dilution and plating onto MRS agar after 0, 3 and 6 h incubation.

### 2.5. Haemolytic Activity

Haemolytic activity of LAB strains was determined according to the method described by Maragkoudakis *et al.* [25] with slight modification. The isolates were grown overnight in MRS broth and then streaked onto Columbia blood agar plates (Fort Richard, Auckland, New Zealand), containing 5% sheep blood. The plates were incubated at 37 °C for 24 h in anaerobic jars. The strains were characterized as haemolytic, partial haemolytic or non-haemolytic depending on the colour change of the agar underlying the colonies. *Listeria monocytogenes* and *Salmonella menston* were used as positive controls. The assay was performed in triplicate.

### 2.6. Antibiotic Resistance

Antibiotic susceptibility was determined by the disc diffusion method. The procedure was adapted from Thirabunyanon *et al.* [26]. Antibiotics tested included (i) inhibitors of bacterial cell wall synthesis: ampicillin 10 μg, amoxicillin 30 μg, vancomycin 30 μg; (ii) inhibitors of protein synthesis: tetracycline 30 μg, chloramphenicol 30 μg, streptomycin 10 μg, gentamycin 10 μg, fusidic acid 10 μg, erythromycin 15 μg; (iii) inhibitors of nucleic acid synthesis: ciprofloxacin 5 μg, nalidixic acid 30 μg. The above antibiotic concentrations are per disc.

### 2.7. Antimicrobial Activity

The antimicrobial activity was determined by the well diffusion assay. The test was carried out according to the method described by Vinderola *et al.* [27], with slight modification. The lactobacilli isolates were cultured in MRS broth overnight and the pathogens were grown in Brain Heart Infusion (BHI) broth (Oxoid, UK). 200 μL of the test pathogens were spread onto the surface of nutrient agar plates. Wells were punctured into the media. 100 μL of CFS (cell free supernatant) obtained by centrifugation of the culture at 13,000 rpm for 1 min (using Microcentrifuge MiniSpin^®^, Eppendorf, Hamburg, Germany), and pH adjusted between 6 and 6.4 was added into the wells. The plates were left inside the refrigerator for 30 min and then incubated at 37 °C for 24 h. The antimicrobial activity of the lactobacilli was determined in terms of development of inhibition zones around the wells. The pathogens tested included *Escherichia coli*, *L. monocytogenes*, *Staphylococcus aureus*, *S. menston* and *Enterobacter aerogenes*.

### 2.8. Assessment of Bacterial Hydrophobicity

The BATH test was employed, to investigate the effect of pH on the hydrophobic nature of the lactobacilli isolates. The isolates were cultured in MRS broth (pH 6.4) overnight at 37 °C. The cells were then centrifuged and washed twice in 1× phosphate buffered saline. A 20 mL suspension was prepared and initial optical density (O.D.) adjusted to an absorbance of 1 at 600 nm. In brief, 6 mL of a suspension of lactobacilli in phosphate buffered saline at pH 1.0, 5.0 and 7.4 adjusted with 1 M HCl was added to 0.7 mL of organic phase (dichloromethane) in glass test tubes (with a tapered bottom) and then vortexed for 5 min. After equilibration for 15 min at room temperature (22 °C) to allow for phase separation, 1 mL of the aqueous phase was transferred to a cuvette without disturbing the organic phase and the O.D. was measured at 600 nm. All experiments were performed in triplicate.

### 2.9. Statistical Analysis

A one-way analysis of variance (ANOVA) was employed, using the program IBM SPSS Statistics (version 21, IBM, Armonk, NY, USA) to evaluate the experimental data for BATH/adherence test. The significant differences were accepted at *p* < 0.05 by Duncan’s test.

## 3. Results

### 3.1. Isolation, Phenotypic Characterization and Carbohydrate Fermentation Profile of Strains

All isolates appeared as round, opaque, creamy or milky white colonies on the surface of MRS agar. When viewed under the microscope after gram staining, they appeared as purple colour rods, suggesting gram positive bacteria. All isolates showed a negative result when tested for oxidase activity. Glucose, galactose, lactose, melibiose, maltose, raffinose, ribose and sucrose were fermented by all isolates. Isolate RC 25 was however incapable of fermenting arabinose. Dairy isolates (MI 6, MI 7 and MI 10) did not ferment cellobiose, mannose, mannitol and sorbitol. Only two isolates, MI 6 and MI 7, were unable to ferment fructose. All isolates tested negative for glycerol utilization. Rumen isolates displayed better capabilities in comparison to dairy isolates, with regard to utilization of available sugars (Table 1).

### 3.2. Identification of LAB

Agarose gel electrophoresis identified DNA bands corresponding to the primers 16-1A and 23-1B. Sequence comparison using BLASTN nucleotide database from the National Center for Biotechnology Information (NCBI) (confirmed that all isolates belonged to species of *Lactobacillus* (Table 2).

### 3.3. Acid Tolerance

**Figure 1:**
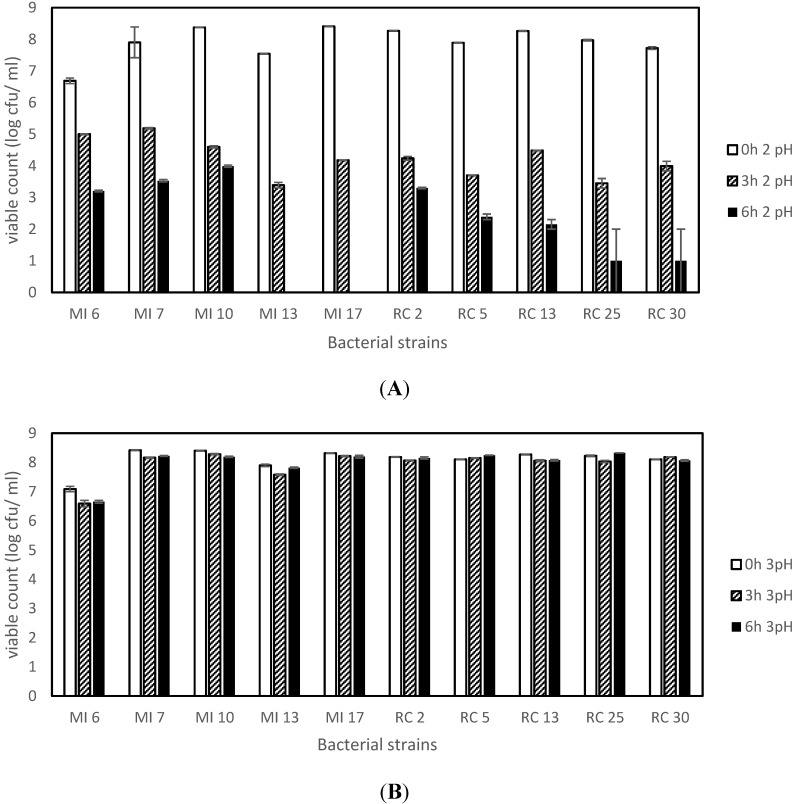
Viability of the strains expressed as log cfu/mL after 0, 3 and 6 h incubation at pH 2 (**A**) and pH 3 (**B**).

At pH 2, the viability of isolates decreased after 6 h, with bacterial isolates MI 13 and MI 17 recording total absence of growth. Dairy isolate, MI 10 recorded maximum tolerance to pH 2 even after an exposure of 6 h. However, with pH 3, the viability was constant in all isolates even after 6 h (Figure 1). Overall, the dairy isolates recorded a slightly increased tolerance to acidic environment, in comparison to rumen isolates.

**Table 1:** Sugar fermentation capacity of each strain.

Strain	Source	Arabinose	Cellobiose	Fructose	Glucose	Galactose	Glycerol	Lactose	Mannose	Mannitol	Melibiose	Maltose	Raffinose	Ribose	Sorbitol	Sucrose
MI 6	dairy food	++	−	−	++	++	−	++	−	−	++	++	++	+	−	++
MI 7	dairy food	++	−	−	++	++	−	++	−	−	++	++	++	+	−	++
MI 10	dairy food	++	−	+	++	++	−	++	−	−	++	++	++	+	−	++
MI 13	dairy food	++	++	++	++	++	−	++	++	++	++	++	++	++	+	++
MI 17	dairy food	++	++	++	++	++	−	++	++	++	++	++	++	++	++	++
RC 2	animal rumen	++	++	++	++	++	−	++	++	++	++	++	++	++	++	++
RC 5	animal rumen	++	++	++	++	++	−	++	++	++	++	++	++	++	++	++
RC 13	animal rumen	++	++	++	++	++	−	++	++	++	++	++	++	++	++	++
RC 25	animal rumen	−	++	++	++	++	−	++	++	++	++	++	++	++	++	++
RC 30	animal rumen	++	++	++	++	++	−	++	++	++	++	++	+	+	++	++

++/+, positive test and −, negative test.

**Table 2:** Strain identification.

Sl. No	Microbial ID	Source	Genetic Identification
1	MI 6	dairy food (yoghurt)	*Lactobacillus reuteri* TD1, complete genome
2	MI 7	dairy food (yoghurt)	Lactobacillus reuteri JCM 1112 DNA, complete genome
3	MI 10	dairy food (yoghurt)	*Lactobacillus reuteri* strain C16
4	MI 13	dairy food (cheese)	*Lactobacillus rhamnosus* LOCK 908, complete genome
5	MI 17	dairy food (cheese)	*Lactobacillus rhamnosus* LOCK 908, complete genome
6	RC 2	animal rumen (cow)	*Lactobacillus plantarum* 16, complete genome
7	RC 5	animal rumen (cow)	*Lactobacillus plantarum* 16, complete genome
8	RC 13	animal rumen (cow)	*Lactobacillus plantarum subsp. plantarum* ST-III, complete genome
9	RC 25	animal rumen (cow)	*Lactobacillus plantarum* 16, complete genome
10	RC 30	animal rumen (cow)	*Lactobacillus plantarum subsp. plantarum* ST-III, complete genome

### 3.4. Bile Salt Tolerance

Rumen isolate RC 25, in particular demonstrated the highest tolerance to 2% of bile salts even after 6 h. On the other hand, dairy isolate MI 13 exhibited the least tolerance after a 6 h period. In general the rumen isolates however, showed a greater tolerance to bile salts after 6 h in comparison to dairy isolates (Table 3).

**Table 3:** Strain viability after 0, 3 and 6 h incubation in 0.3% or 2% bile salts.

Strain	Source	0.3% Bile			2% Bile		
		0 h	3 h	6 h	0 h	3 h	6 h
MI 6	dairy food	9.794 ± 0.054	8.016 ± 0.088	8.777 ± 0.087	9.658 ± 0.007	9.078 ± 0.051	9.078 ± 0.051
MI 7	dairy food	10.021 ± 0.012	8.574 ± 0.008	8.562 ± 0.008	10.158 ± 0.013	8.889 ± 0.157	9.230 ± 0
MI 10	dairy food	9.602 ± 0	8.984 ± 0.003	8.469 ± 0.031	9.511 ± 0.113	9.102 ± 0.144	8.778 ± 0.249
MI 13	dairy food	9.451 ± 0.213	8.067 ± 0.021	9.434 ± 0.025	7.827 ± 0.181	8.661 ± 0.260	7.389 ± 0.125
MI 17	dairy food	8.866 ± 0.125	9.075 ± 0.010	8.396 ± 0.049	8.651 ± 0.069	8.540 ± 0.088	7.500 ± 0.281
RC 2	animal rumen	9.217 ± 0.019	9.428 ± 0.017	9.477 ± 0	8.923 ± 0.110	9.289 ± 0.047	9.755 ± 0.043
RC 5	animal rumen	10.069 ± 0.013	9.041 ± 0.017	9.477 ± 0	10.234 ± 0.009	9.136 ± 0.134	9.581 ± 0.088
RC 13	animal rumen	9.413 ± 0.047	9.118 ± 0.012	9.346 ± 0.008	9.802 ± 0.044	8.690 ± 0.125	9.711 ± 0.042
RC 25	animal rumen	8.922 ± 0.110	8.841 ± 0.013	9.477 ± 0	9.589 ± 0.063	9.300 ± 0.031	9.920 ± 0.048
RC 30	animal rumen	9.096 ± 0.025	8.679 ± 0.051	9.229 ± 0.033	9.871 ± 0.037	9.918 ± 0.052	9.590 ± 0.032

Presented values are means of duplicate determinations. ± indicates standard deviation from the mean.

### 3.5. Haemolytic Activity

All ten lactobacilli isolates tested negative for haemolytic activity. The positive controls, *L. monocytogenes*, exhibited complete haemolysis and *S. menston* exhibited partial haemolysis (data not shown).

### 3.6. Antibiotic Resistance

The isolates showed 100% resistance to streptomycin, gentamycin, ciprofloxacin, vancomycin, kanamycin and nalidixic acid. Four dairy isolates and a rumen isolate showed resistance to tetracycline, four rumen isolates showed intermediate resistance and one dairy isolate was susceptible to the tetracycline. Three dairy isolates were resistant to erythromycin, five rumen isolates and one dairy isolate showed intermediate resistance and one dairy isolate was susceptible to the erythromycin. Maximum susceptibility was observed with chloramphenicol and ampicillin with all rumen and two dairy isolates being susceptible and three dairy isolates showing resistance (Table 4). In general dairy isolates exhibited a better antibiotic resistance profile than rumen isolates.

**Table 4:** Strain antibiotic resistance profile against various antibiotics tested.

Strain	Source	Antibiotic Resistance *										
		TE	ST	NA	CN	FA	VA	K	C	E	CIP	AMP
MI 6	dairy food	R	R	R	R	R	R	R	R	R	R	R
MI 7	dairy food	R	R	R	R	R	R	R	R	R	R	R
MI 10	dairy food	R	R	R	R	R	R	R	R	R	R	R
MI 13	dairy food	S	R	R	R	R	R	R	S	S	R	S
MI 17	dairy food	R	R	R	R	R	R	R	S	I	R	S
RC 2	animal rumen	R	R	R	R	R	R	R	S	I	R	S
RC 5	animal rumen	I	R	R	R	R	R	R	S	I	R	S
RC 13	animal rumen	I	R	R	R	R	R	R	S	I	R	S
RC 25	animal rumen	I	R	R	R	R	R	R	S	I	R	S
RC 30	animal rumen	I	R	R	R	R	R	R	S	I	R	S

AMP = ampicillin, ST = streptomycin, CIP = ciprofloxacin, VA = vancomycin, C = chloramphenicol, CN = gentamycin, NA = nalidixic acid, E = erythromycin, TE = tetracycline, FA = fusidic acid, K = kanamycin. * R = Resistant, S = Sensitive and I = Intermediate resistance.

### 3.7. Antimicrobial Activity

None of the isolates could inhibit the growth of *E. coli* on the nutrient agar plates. Maximum size of inhibition zones was exhibited against *L. monocytogenes.* A mixed response was seen in case of *E. aerogenes*, *S. aureus* and *S. menston*. Three dairy isolates MI 6, MI 7 and MI 10, did not show any inhibitory effect on *Listeria* species (Table 5). Rumen isolates portrayed better antimicrobial activity towards the pathogens.

**Table 5:** Antimicrobial activity profile of strains against various pathogens.

Strain	Source	*Escherichia coli*	*Listeria monocytogenes*	*Enterobacter aerogenes*	*Staphylococcus aureus*	*Salmonella menston*
MI 6	dairy food	−	−	±	±	+
MI 7	dairy food	−	−	±	+	+
MI 10	dairy food	−	−	±	±	±
MI 13	dairy food	−	+	−	+	±
MI 17	dairy food	−	+++	±	±	+
RC 2	animal rumen	−	+	±	+	+
RC 5	animal rumen	−	++	+	−	+
RC 13	animal rumen	−	+++	+	±	+
RC 25	animal rumen	−	+++	+	+	+
RC 30	animal rumen	−	+++	+	+	+

Zone of inhibition <0 mm (−), 0–4 mm (±), 4–8 mm (+), 8–12 mm (++) and >12 mm (+++).

### 3.8. Bacterial Adherence to Hydrocarbons

**Figure 2:**
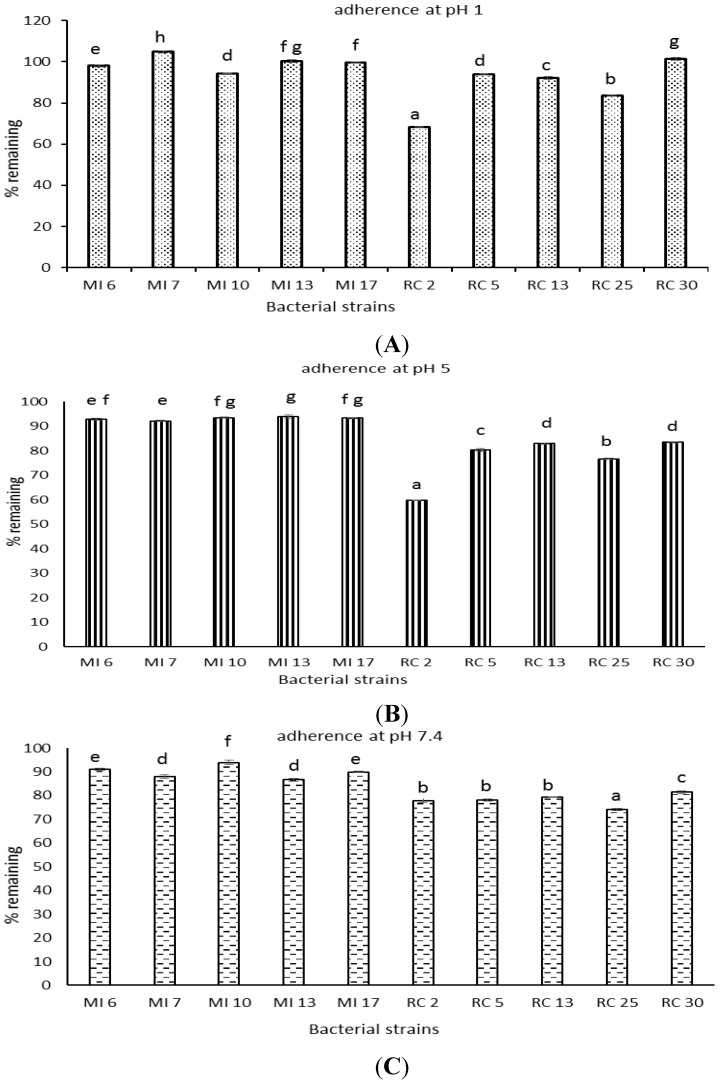
Percent initial OD at 600 nm following lactobacilli strains adherence at pH 1 (**A**); pH 5 (**B**); and pH 7.4 (**C**) to dichloromethane. Standard error of the mean bars (*n* = 3) and columns with different letters for a given pH differ significantly (*p* < 0.05).

The adsorbence of the lactobacilli isolates to dichloromethane showed an isolate and pH effect. At lower pH the rumen isolates were more adsorbent to the dichloromethane. Maximum adherence was exhibited between pH 1–pH 5. Rumen isolates RC 2 and RC 25 showed maximum absorbance to dichloromethane (Figure 2).

## 4. Discussion

Screening of lactobacilli from diverse ecological niches for potential probiotic applications has been systematically carried out. However, a comparative profile of lactobacilli from two different ecological niches has not been reported. So, the focus of our study was to test the dairy and rumen isolates under similar challenges and compare their results to see which group of isolates performed better. An ideal animal rumen pH is 6–7. All rumen isolates are well adapted to grow and survive in the ruminant digestive system where it is exposed to range of stressful conditions in the GIT. However, their challenge would lie in growing under human GIT conditions. Having a common experimental design for dairy and rumen isolates was necessary to compare and interpret their results. Rumen isolates may be exposed to antibiotics through their clinical use as animal health therapeutics. One such example includes a condition known as postpartum metritis in cows. Antibiotics have typically performed poorly when treating this condition however traces of antibiotics can be found in milk [28,29]. This suggests that rumen isolates might from time to time be exposed to therapeutic antibiotic use and it could be expected that possession of non-transferable resistance would be a beneficial characteristic and aid *in vivo* survival. All the lactobacilli isolates used in this study, which include species—*reuteri*, *rhamnosus* and *plantarum*, are GRAS (Generally Regarded As Safe) according to the New Zealand Agricultural Compounds & Veterinary Medicines (ACVM) group of the MPI (Ministry for Primary Industries). Till date, there have been no reported incidents of pathogenicity associated with the use of these bacteria [30]. It is expected that the ten isolates used in this study could be used as potential probiotics for humans, poultry or livestock. They can either be incorporated into foods for human consumption, imparting a general overall well-being to the host or they could also be used in animal feed for protection against infections or as growth promoters. In this study, all dairy and rumen isolates were subjected to a range of physiologically applicable stresses and *in vitro* responses were analysed.

Firstly, the ten isolates used in this study were identified by phenotypic characterization and genotypic methods. After being identified as lactobacilli, they were subjected to screening for potential probiotic abilities. All the isolates proved capable of tolerating low pH conditions (pH 2 and pH 3). However, viability decreased with a decrease in pH. In similar studies, it was found that the strains could tolerate and survive in MRS broth of pH 3, whereas low viability was observed at pH 2 [31,32]. Bile salt at a concentration of 0.3% is the maximum that can be found in an average healthy person. Thus, in this study 0.3% was selected as the starting range for screening the isolates. The result showed that all isolates could tolerate the 0.3% and 2% bile salt condition. However, higher tolerance was monitored at 0.3% concentration of bile salt. Our findings are similar with those reported elsewhere where lactobacilli was found to grow well and multiply in 0.3% of bile salt [33]. As per safety concerns, a potential probiotic bacteria should not cause lysis of red blood cells in the body. *In vitro* investigation of this was done by testing the isolates for haemolytic activity; lactobacilli are usually non-haemolytic in nature. All the ten isolates were incapable of exhibiting haemolysis on the agar media containing 5% sheep blood. This is in agreement with other reports of lactic acid bacteria and bifidobacteria species confirming that they are non-haemolytic in nature [34]. Regarding antibiotic resistance, the isolates showed a mixed response. All ten isolates showed resistance to streptomycin, nalidixic acid, gentamycin, fusidic acid, vancomycin, kanamycin and ciprofloxacin. In case of tetracycline, four dairy isolates (MI 6, MI 7, MI 10, and MI 17) and one rumen isolate (RC 2) showed resistance. One dairy isolate, MI 13, was sensitive to four antibiotics. Four rumen isolates, RC 5, RC 13, RC 25 and RC 30, showed intermediate resistance towards the antibiotic. For erythromycin; three dairy isolate (MI 6, MI 7, MI 10) showed high resistance, one dairy isolate, MI 13, was sensitive and remaining six isolates showed intermediate resistance. Maximum susceptibility was observed against chloramphenicol and ampicillin. In some lactobacillus species, such as *L. rhamnosus*, *L. casei*, *L. plantarum*, *L. fermentum*, *L. brevis*, and *L. curvatus*, vancomycin resistance has been reported as an inherent property of the strain that is not re-transferable across species or genus [35]. Thus resistance observed against vancomycin in this study was not unexpected. Previous studies also noted a high resistance to aminoglycosides such as kanamycin, streptomycin and gentamicin amongst lactobacilli [22,36]. Likewise previous studies have also reported high resistance to nalidixic acid [7]. Studies have reported that lactobacilli are usually sensitive to ampicillin [37,38]. Innate resistance of probiotics to some antibiotics suggests their use for preventive and therapeutic purposes in controlling intestinal infections especially when co-administered with the therapeutic use of antibiotics. According to the antimicrobial activity data obtained, it was observed that all ten isolates did not inhibit *E. coli*. Four rumen isolates, RC 5, RC 13, RC 25 and RC 30, were able to inhibit *E. aerogenes*. Only MI 13 was incapable of inhibiting *E. aerogenes*. Growth of *S. aureus* was inhibited by MI 7, MI 13, RC 2, RC 25 and RC 30. Rumen isolate RC 5 did not inhibit *S. aureus*. *S. menston* inhibition was observed by all five rumen isolates and three dairy isolates, MI 6, MI 7 and MI 17. The highest degree of inhibition was observed against *L. monocytogenes*, where all five rumen isolates and two dairy isolates MI 13 and MI 17 showed clear inhibition zones. Inhibition of gram-positive bacteria, such as *L. monocytogenes* and *S. aureus*, by lactobacillus species has been described previously [20]. We found that rumen isolates tended to inhibit the growth of these pathogens to a greater extent than the isolates form dairy sources. This is perhaps not unexpected, as the rumen isolates would possibly co-exist with these pathogens or at the least have to compete *in vivo* during incidences of infection by these pathogens of the host form, which the isolates were collected. Rumen isolate RC 2 showed the maximum adherence per cent at pH 1 and pH 5, closely followed by the rumen isolate RC 25. However, at pH 7.4 RC 25 showed the highest adherence followed by RC 2. Overall the data suggests that the dairy isolates had very poor adherence properties in comparison to the rumen isolates. Cell surface hydrophobicity as an indication of potential adherence capabilities is an important characteristic of potential probiotics. Strains with a good adherence property indicate that they might be better capable of binding to the intestinal epithelial lining and improving the cell barrier functions [39]. This mechanism is one of the major factors by which probiotic bacteria are believed to exert beneficial effects in the host.

## 5. Conclusions

In conclusion, this study indicates that isolates of rumen origin exhibited a slightly increased tolerance to adverse stress conditions, especially towards presence of bile salts, inhibition of pathogens and adherence property, in comparison to isolates of dairy origin. This study has provided valuable information on the *in vitro* characteristics of rumen and dairy isolates, which has helped in the identification of potential probiotic candidates that can be used for further investigation and development as potential probiotics in foods and complementary and alternate medicines.
